# The psychological profile of patients with hypertension and its role in patients’ self-care

**DOI:** 10.1192/j.eurpsy.2024.1026

**Published:** 2024-08-27

**Authors:** T. Stamoulis, E. Dragioti, M. Gouva, M. Kourakos

**Affiliations:** ^1^Research Laboratory Psychology of Patients, Families and Health Professionals, Department of Nursing, School of Health Sciences, University of Ioannina, Ioannina, Greece

## Abstract

**Introduction:**

Hypertension is a major risk factor for premature death, cardiovascular disease, and stroke worldwide. However, due to the chronic nature of hypertension, patients are at an increased risk of developing mental and emotional disorders, which can affect their adherence to self-care.

**Objectives:**

To conduct a systematic review to investigate the psychological profile, psychological characteristics, and personality traits of hypertensive patients and their role in self-care adherence.

**Methods:**

A thorough and comprehensive literature search was conducted to identify relevant studies for this review. PubMed, Scopus, and APA PsycInfo databases were searched from their inception until March 7th, 2023. The protocol for this review will be registered in the International Prospective Register of Systematic Review (PROSPERO) in the future.

**Results:**

After applying inclusion and exclusion criteria and removing duplicate results, 55 articles were selected, the majority of which were grouped into three main categories based on psychological profiles for accurate analysis and comparison.

**Conclusions:**

This systematic review contributes to the investigation of the relationship between psychological profiles and self-care in hypertensive patients. Despite the prevailing controversy in the literature, a greater proportion of studies indicate that depression, anxiety, low quality of life, type D personality and neurotic personality have a negative impact on self-care in hypertension.

**Disclosure of Interest:**

None Declared

